# An analysis of large structural variation in global *Plasmodium falciparum* isolates identifies a novel duplication of the chloroquine resistance associated gene

**DOI:** 10.1038/s41598-019-44599-0

**Published:** 2019-06-04

**Authors:** Matt Ravenhall, Ernest Diez Benavente, Colin J. Sutherland, David A. Baker, Susana Campino, Taane G. Clark

**Affiliations:** 1 Department of Pathogen Molecular Biology, London School of Hygiene and Tropical Medicine, London, WC1E 7HT UK; 2 Department of Immunology and Infection, London School of Hygiene and Tropical Medicine, London, WC1E 7HT UK; 3 Department of Infectious Disease Epidemiology, Faculty of Epidemiology and Population Health, London School of Hygiene and Tropical Medicine, London, WC1E 7HT UK

**Keywords:** Genome informatics, Malaria

## Abstract

The evolution of genetic mechanisms for host immune evasion and anti-malarial resistance has enabled the *Plasmodium falciparum* malaria parasite to inflict high morbidity and mortality on human populations. Most studies of *P. falciparum* genetic diversity have focused on single-nucleotide polymorphisms (SNPs), assisting the identification of drug resistance-associated loci such as the chloroquine related *crt* and sulfadoxine-pyrimethamine related *dhfr*. Whilst larger structural variants are known to impact adaptation, for example, *mdr1* duplications with anti-malarial resistance, no large-scale, genome-wide study on clinical isolates has been undertaken using whole genome sequencing data. By applying a structural variant detection pipeline across whole genome sequence data from 2,855 clinical isolates in 21 malaria-endemic countries, we identified >70,000 specific deletions and >600 duplications. Most structural variants are rare (48.5% of deletions and 94.7% of duplications are found in single isolates) with 2.4% of deletions and 0.2% of duplications found in >5% of global isolates. A subset of variants was present at high frequency in drug-resistance related genes including *mdr1*, the *gch1* promoter region, and a putative novel duplication of *crt*. Regional-specific variants were identified, and a companion visualisation tool has been developed to assist web-based investigation of these polymorphisms by the wider scientific community.

## Introduction

*Plasmodium falciparum* malaria imposes a heavy morbidity and mortality burden, with an estimated 216 million new cases and 446,000 deaths in 2016 alone, with ~90% of the burden in sub-Saharan Africa^1^. An understanding of the genomic diversity of *P. falciparum* parasites could provide insights into novel phenotypes that impact responses to antimalarials and other control measures, as well as host-pathogen interactions. Single nucleotide polymorphism (SNP) based analyses have revealed insights into drug resistance, molecular barcodes for continental origin^2^, transmission dynamics^3^, multiplicity of infection^4^ and regions under selective pressure related to immunological and anti-malarial treatment pressure^5^. In comparison, investigations of structural variants (SVs), such as insertions, deletions and duplications, have been sparse. This is despite SVs making an important contribution to genomic diversity and comprising many nucleotides of heterogeneity. In particular, copy number variants (CNVs; large indels and duplications) are thought to be widespread in the *P. falciparum* genome^6^.

Malaria parasites are exposed to strong selection from the human immune response and treatment with antimalarial drugs. Subsequently, CNVs have often been found in association with specific *P. falciparum* phenotypes, such as drug resistance. Duplications of *mdr1* have been shown to underlie a multi-drug resistance phenotype, with these variants now present at high population frequencies in Southeast Asia^7^, with copy number altering the parasite response to multiple anti-malarial drugs^8^. Recently, we identified a novel promoter duplication for *gch1* at near-fixation in a Malawi population^5^, which is distinct from the whole gene duplication observed in Southeast Asia known to contribute to sulfadoxine-pyrimethamine (SP) resistance. In general, such regional genetic variation may arise from differences in drug regimens, mosquito vectors, and host immunity, but is poorly understood.

Given their importance, a genome-wide structural variant map for *P. falciparum* with country and regional resolution should provide insights with which to better understand the impacts of treatment regimes, assess changes in parasite diversity, and ultimately inform the roll-out of anti-malarial drugs and other control initiatives. The advent of microarray technologies, such as genomic hybridisation arrays (CGH), has improved methods for detecting and confirming known SVs^9,10^. However, studies of this type have typically featured modest sample sizes and focused on the exome of lab-adapted isolates. The largest array-based study in *P. falciparum* clinical isolates (n = 122) identified 134 high-confidence CNVs across the parasite exome, established they were more common in South American than African or Southeast Asian populations, and identified several loci including *mdr1, rh2b*, and histidine-rich proteins II and III to be under positive selection^10^. Recently whole genome sequencing platforms, which produce a greater depth of short or long reads, have been used to detect SVs in *P. falciparum* strains^11,12^, and have potentially finer resolution than array-based approaches. Coupled with bioinformatic advances in detection algorithms, there is now capacity to accurately characterise a broader range of SV types. For example, extremes in coverage can identify duplications and deletions, split sequences and alternative *de novo* assembly-based approaches can detect a number of other types, including inversions and large insertions and deletions^13^.

By analysing whole genome sequencing data from 2,855 clinical isolates and focusing on robust genomic regions (82.6%) of the AT-rich *P. falciparum* genome, we present the first comprehensive genomic map of SVs within the global *P. falciparum* population, with a focus on CNVs. We identify a total of 70,257 high quality deletions (mean 440.56 per isolate, median size 26 bp) and 601 high quality duplications (mean 0.43 per isolate, median size 1,478 bp), contrasting with an average of 24,495 SNPs and 33,479 small indels (<15 bp) per isolate. Several variants were found to be geographically specific and highlight novel structural variants with roles in antigenic variation, drug resistance, and host-pathogen interactions. We confirm specific variants using several alternative approaches, including the analysis of PacBio long-read data of *P. falciparum* laboratory strains.

## Results

### Distribution of variant type, size and location

**Figure 1:**
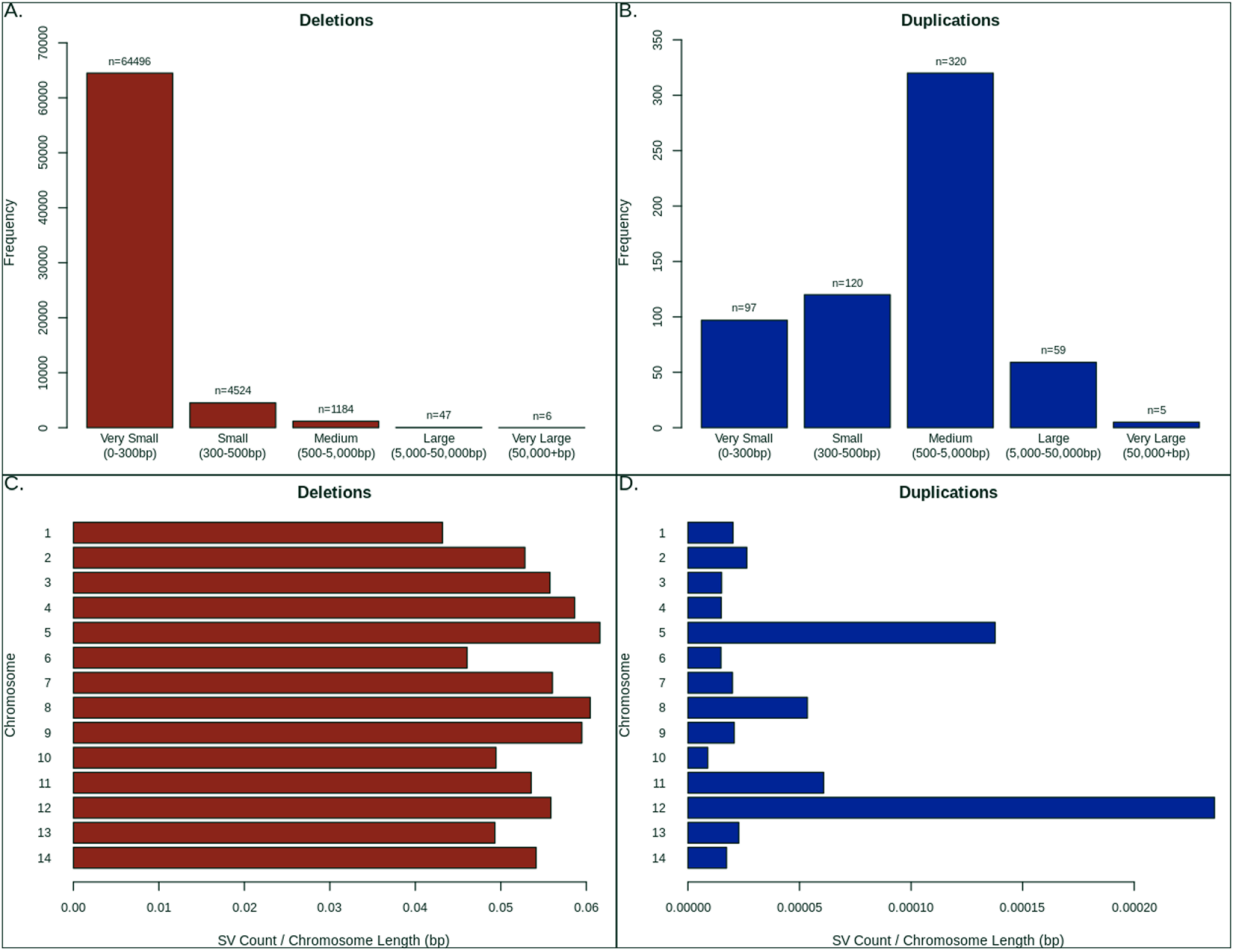
High quality variants by position, length and per-chromosome. (**A**) Distribution of deletions by size categories. (**B**) Distribution of duplications by size categories. (**C**) Distribution of distinct form of deletion across each chromosome. (**D**) Distribution of distinct forms of duplication across each chromosome.

The 2,855 isolates represented 21 countries across Africa (Central, East, West), Asia (South, Southeast) and South America (Supplementary Table 1), and all displayed minimal evidence of multiplicity of infection (based on genome-wide SNPs) and non-anomalous coverage (see Methods). Using a structural variant (SV) discovery pipeline based primarily upon DELLY^13^, we identified more than 1 million putative variants deletions and duplications relative to the 3D7 reference genome, across robust regions (~83%; excluding regions that were highly variable or within 100 kbp of a chromosome end) of the *P. falciparum* genome. SVs of length greater than 300 bp were present in 4,941 genes, including in known drug resistance candidates (e.g. *crt, mdr1, gch1*) and indel/duplication hotspots, such as within the liver stage antigen *LSA1*^14^, the gametocyte specific *Pf11-1*^14^ and invasion-related *Rh2b*^15^ and *EBA175* (*PF3D7_0731500*)^16^ genes (see Supplementary Table 2 for the 117 genes with >1% SV frequency). This raw set was filtered using a population-based SV analysis pipeline (see Methods), and the resulting dataset of 70,858 ‘high-quality’ variants included 70,257 deletions (mean 440.56 per isolate, median size 26 bp; 91.8% very small or micro <300 bp) and 601 duplications (mean 0.43 per isolate, median 1,478 bp; 16.1% very small or micro <300 bp). Most duplications (94.7%) and half of deletions (34,065 deletions; 48.6%) were unique to single isolates (total: 34,634; 34,065 deletions and 569 duplications) (Fig. 1). Both deletions and duplications tend to occur within intergenic regions (intergenic/genic ratio: deletions 1.42, duplications 2.15), and there is disproportional increase in the density of duplications in chromosomes 5 and 12, due to known drug resistance loci (*mdr1, gch1*) (Fig. 1). The most frequent genes with high-quality SVs (>300 bp) reveals loci that include both deletions and duplications (e.g. *LSA3*), and clusters of duplications (e.g. in chromosome 11: *FP3, ApiAP2, CYP19B, FP2A, TREP*) (Supplementary Table 3). Therefore, a (1 kbp) window-based analysis was used to identify regions with overlapping but distinct SVs, thereby assisting with refining their breakpoints. For deletions, 24,947 (of 27,388) windows (78.1% genic) were represented, compared to 2,441 windows (80.4% genic) for duplications.

### Frequently occurring specific variants

Previous work has shown that SVs present in a relatively high proportion of the global population are consistent with evidence of phenotypic selection^10^, we therefore prioritised common variants, that is, present in at least 1% of our isolates (29 or more). In total we identify 7,618 common variants of which only two are duplications, one being the previously identified 436 bp *gch1* promoter region duplication^5^ and the other being a 252 bp intergenic duplication (11:1029648–1029899; between *PF3D7_1126300* and *PF3D7_1126400*) and exclusive to Southeast Asia. Of all common variants, 2,780 (36.5%) are genic and the median length is 25 bp (see Supplementary Table 4 for the subset with global frequency >35%). At a minimum frequency of 5%, there were 1,676 variants with median length 27 bp, including 723 (43.1%) genic and 1 duplication (*gch1*). We focused primarily on SVs in excess of 300 bp in length, as DELLY calling is considered more reliable at this threshold^13^. Only 36 loci or regions with common variants greater than 300 bp in length (Supplementary Table 5) were identified, including four intergenic deletions (size range: 605 to 1,023 bp), and one 553 bp deletion in *LSA3* (*PF3D7_0220000*). Interestingly the 553 bp deletion in *LSA3* is present primarily in Southeast Asia, particularly Thailand (14.6%), Laos (11.5%), and Myanmar (11.2%) (Global 5.5%; Africa 0.1%, America 0.0%, Asia 10.2%), and may represent region-specific host-directed selection. Two of the intergenic variants show evidence of continental differences (Allele frequency difference: F_ST_ score >0.2), including a 1,015 bp deletion in chromosome 9 upstream of *gexp22* (*PF3D7_0935500*) (F_ST_: 0.227; Africa 13.2%, America 70.8%, Asia 41.7%) and a 605 bp deletion in chromosome 12 upstream of *ap2mu* (*PF3D7_1218300*) (F_ST_ 0.249; 0.2% Africa, 0.0% America, 33.6% Asia).

### Exploration of structural variation in anti-malarial resistance candidates

**Figure 2:**
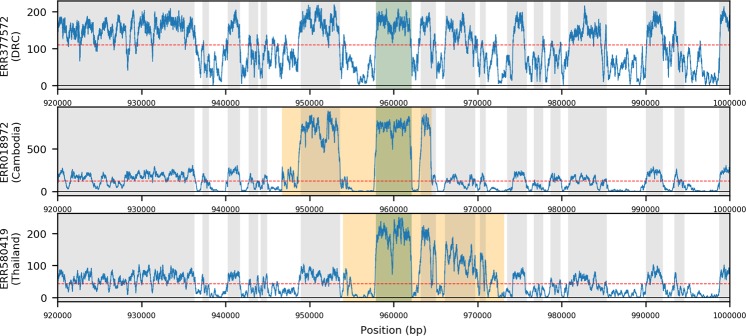
Coverage plots showing examples of isolates with three types of *mdr1* duplications. (**A**) No duplication in a Democratic Republic of Congo isolate. (**B**) Duplication in a Cambodian isolate. (**C**) Duplication in a Thai isolate; Blue traces represents the per base coverage for each isolate. Orange region indicates the predicted structural variant; Green region indicates the gene of interest, Grey indicates neighbouring genes; horizontal line is the median coverage for the isolate.

Previous analyses have revealed evidence of structural variation in loci associated drug resistance (e.g. *gch1*). Given that SVs can have a significant impact on gene expression and anti-malarial resistance, we focused an analysis on the identification of novel structural variants in candidate genes (*dhfr*, *dhps*, *kelch13, mdr1, gch1, crt*). Despite removing mixed infections determined using genome-wide SNPs, it is possible for heterogeneous duplications (i.e. differences in genetic copies) to display as mixed genotype calls, therefore we extended our ‘high-quality’ dataset to include those duplications previously excluded for high rates of the heterozygous genotype. The resulting ‘high-quality relaxed’ dataset included 91,936 putative variants (70,257 deletions: mean 440.56 per isolate, median size 26 bp; 21,679 duplications: mean 10.92 per isolate, median 1,354 bp). To minimise the number of false positives, we manually verified the alignments for all candidate regions specifically mentioned here. Overall, no high-quality SVs were identified in SP resistance associated *dhfr* (*PF3D7_0417200*) or *dhps* (*PF3D7_0810800*) genes, or artemisinin resistance associated *kelch13* (*PF3D7_1343700*). We identify 115 specific duplication types containing *mdr1* in 189 isolates, primarily in Southeast Asia (Southeast Asia 12.9%; Cambodia 9.5%, Myanmar 11.9%, Papua New Guinea 3.8%, Thailand 29.0%, Vietnam 5.9%), and near absent in Africa (Africa 0.10%; Ghana 0.2%) (Fig. 2), consistent with previous reports^17^. Similarly, tandem duplications are also present in KE01 (Kenya) and KH01 (Cambodia) within our complementary PacBio-based dataset (n = 13).

**Figure 3:**
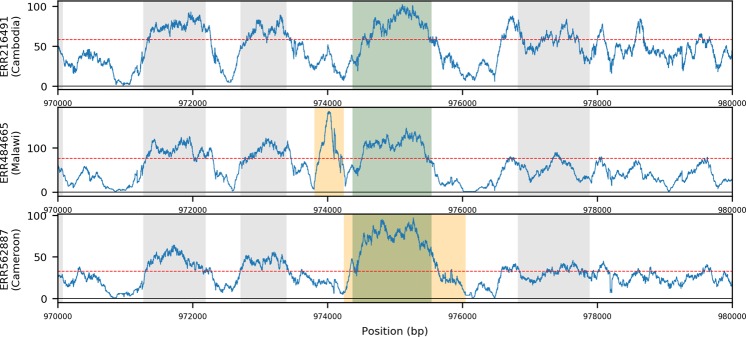
Coverage plots showing examples of isolates with three types of *gch1* duplications. (**A**) No duplication in a Cambodian isolate. (**B**) Promoter duplication in a Malawian isolate. (**C**) Gene Duplication in a Cameroon isolate; Blue traces represent the per base coverage for each isolate. Orange region indicates the predicted structural variant; Green region indicates the gene of interest, Grey indicates neighbouring genes; horizontal line is the median coverage for the isolate.

The whole gene duplication of *gch1* (*PF3D7_1224000*) and a recently identified 436 bp *gch1* promoter duplication may be linked to SP resistance^5^. We identify 307 isolates with 135 distinct forms of whole gene duplication across *gch1* (9.9% of the total dataset) (Fig. 3). Similar whole gene tandem duplications were present in PacBio isolates for 7G8 and KH02, as a triplication in GB4, and as a triplication with an inverted middle copy in DD2; this being consistent with the existing literature^12^. In contrast, 491 high quality isolates are positive for the previously identified 436 bp specific ‘promoter region’ duplication (14.0% of total). We confirm this duplication being present at near-fixation in Malawi (89.5%), frequent in the rest of East Africa (Tanzania 78.5%, Kenya 31.6%), maintained in West Africa (Gambia 6.1%, Ghana 4.3%, Guinea 22.2%) and Central Africa (Democratic Republic of Congo 26.3%), but absent from all Asian and American isolates (Regional F_ST_ 0.554). No such duplication was found in any of the validation isolates with PacBio sequencing data (n = 13), though none of these were isolated from Malawi. These data therefore support the *gch1* promoter duplication being present at notable frequency across Africa, and the need for further functional characterisation of any potential role in SP resistance.

**Figure 4:**
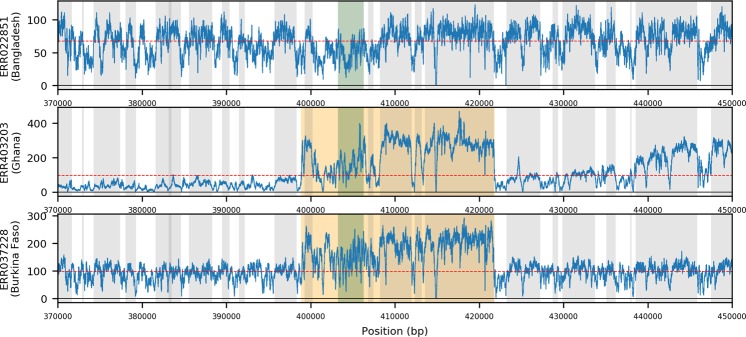
Coverage plots showing examples of isolates with three types of *crt* duplications. (**A**) No duplication in a Bangladesh isolate. (**B**) Gene duplication in a Ghanaian isolate. (**C**) Gene duplication in a Burkina Faso isolate; Blue traces represents the per base coverage for each isolate. Orange region indicates the predicted structural variant; Green region indicates the gene of interest, Grey indicates neighbouring genes; horizontal line is the median coverage for the isolate.

Finally, we uncover evidence of duplications of *crt* (*PF3D7_0709000*) across 35 West African and 2 Cambodian isolates. A 22.9 kbp duplication of *crt* is present and consistent across 32 isolates sourced in West Africa (4.3%), specifically sub-populations isolated in Burkina Faso (n = 14, 29.2%), Ghana (n = 15, 3.4%), Guinea (n = 1, 0.85%) and Mali (n = 1, 1.8%). Those 32 isolates display 26 specific variants, the consensus of which suggests that the duplication is most likely approximately 22,893 bp in length, and therefore includes several genes (*PF3D7_0708900* (*sco1*), *PF3D7_0709000* (*crt*), *PF3D7_0709050* (small nucleolar RNA), *PF3D7_0709100* (*cg1*), *PF3D7_0709200* (*glp3*) and *PF3D7_0709300* (*cg2*)) (Fig. 4). A further three isolates (2 from Ghana, 1 from Burkina Faso) display a similar 28.7 kbp duplication, which also includes *PF3D7_0708800* (heat shock protein 110) and may be under different selective pressure. Beyond Africa, two Cambodian isolates feature smaller 702 bp and 11,844 bp duplications. No *crt* duplication was present in the validation (PacBio) dataset (n = 13). To explore the specific variability of *crt* in West Africa, we calculated the abundance of resistance-associated haplotypes (alleles) directly from raw reads, finding that only both CVMNK (chloroquine susceptible) and CVIET (chloroquine resistant) haplotypes were present in all 35 duplication-positive isolates; this compared to 20.3% (133/656) of isolates with evidence for mixed haplotypes but no evidence of duplication (Supplementary Table 6). Increasing the stringency on the calling of genotypes led to the retention of a disproportional number of mixed haplotypes in those samples with duplications (at least 2 (10) reads required to call haplotypes: duplications 33/35 (24/35) vs. no duplications 99/133 (44/133)). For those isolates with mixed haplotype calls (n = 168), the levels of *crt* gene to chromosome 7-wide coverage were greater in the duplication group (ratio median: duplication group (n = 35) 1.10 vs non-duplication (n = 133) 0.74; Wilcoxon P = 1.7 × 10^−10^). There was no difference in the coverage ratio within the non-duplication group (ratio median: mixed haplotypes (n = 133) 0.74 vs. single haplotype (n = 523) 0.76; Wilcoxon P = 0.09). These observations lend support to the robustness of duplications in *crt*. The proportion of CVIET haplotype reads in the duplication-positive group (median 0.53; IQR: 0.31–0.57; Supplementary Fig. 1) suggested a degree of parity of the carriage of chloroquine susceptible and resistant forms, and contrasted with other West African isolates without duplications (Wilcoxon P = 0.02; Supplementary Fig. 1). It is unclear, without additional transcriptional analysis, whether these forms are expressed independently though we hypothesise that the presence of both forms may allow individual parasites to benefit from the resistance form whilst reducing associated fitness costs. If so, a heterogeneous duplication of this sort may represent a more evolutionarily resilient form of *crt-*associated resistance.

### Population-Specific Variants

Regional differences (across West Africa, Central Africa, East Africa, South Asia, Southeast Asia, South America) in SV frequencies were quantified with F_ST_ analysis for all high-quality variants (median (range): deletions 0.002 (0–0.613); duplications 0.001 (0–0.554)). A total of 153 high quality variants (152 deletions and one duplication) have strong regional differences (F_ST_ > 0.2), including: (i) an Asia-specific 59 bp deletion within the hypothetical protein *PF3D7_0312900* (F_ST_ 0.613, 69.8% South Asia, 70.9% Southeast Asia, 0.0% Rest of the World), (ii) a 40 bp South America-specific deletion in the putative histone deacetylase *PF3D7_1472200* (F_ST_ 0.497, 54.2% South America, 0.0% Rest of the World), and (iii) the 436 bp *gch1* promoter region duplication (F_ST_ 0.554, 78.0% East Africa, 17.2% Central Africa, 6.8% West Africa, 0.0% Rest of the World) (Supplementary Table 7).

Extending our analysis to the full list of SVs detected by the DELLY pipeline that were confirmed using alternative coverage-based approaches (CNVNator or Control-FREEC software), we identity several SVs with biogeographical specificity. Non-drug resistance candidates include a 169 bp deletion within the rhoptry-associated membrane antigen *PF3D7_0707300* (F_ST_ 0.354; 46.0% Africa, 0.6% Asia, 0.0% America) and a 370 bp deletion in the ring-infected erythrocyte surface antigen *PF3D7_0102200* (F_ST_ 0.213; Africa 40.3%, Asia 4.2%, America 4.2%) with elevation in West and Central Africa (F_ST_ 0.321; West Africa 66.1%, Central Africa 36.1%, East Africa 4.1%, South Asia 50.9%, Southeast Asia 2.5%, South America 4.2%). We also identify a near Africa-specific 586 bp deletion within the C-terminal of reticulocyte binding protein 2 homologue b (*rh2b*, PF3D7_1335300) (F_ST_: 0.334; 58.4% Africa, 12.5% America, 1.3% Asia) and a 29 bp deletion in *rhopH2* (PF3D7_0929400) (F_ST_ 0.288; 73.7% Africa, 100% America, 40.8% Asia), knockdown of which has been shown to inhibit parasite growth within host erythrocytes^18^. These findings were supported by manual inspection of coverage depth and split read support.

## Discussion

This large and geographically comprehensive study of SVs in *P. falciparum* characterises both known and novel variants, the latter occurring in loci associated with antimalarial resistance, host-pathogen interactions, and disease severity. Deletions represent the bulk of SVs (>99%) identified, primarily due to an abundance of shorter forms (median 26 bp) in comparison to duplications (median 713 bp). We find that 48.6% of high quality deletions and 65.0% of high quality duplications were found in single isolates, in line with previous work with smaller sample sizes including a recent study which found that approximately half of structural variants were only present in one of 16 isolates^9^. Previous large-scale studies have often overlooked the role of smaller structural variants (15 to 500 bp), defining and applying a minimum size of 500 bp. Our results demonstrate that a significant number (97.7%) of high quality variants are present in the 15 to 500 bp size range, indicating that previous studies may have under-estimated the full range of genomic variants within the *P. falciparum* genome. This finding that most SVs are under 500 bp in size is consistent with previous studies in various species^9,19^.

Population-specific SVs suggest evidence of localised selective pressure^10^. These include the drug resistance associated *mdr1* and *gch1* genes, and a striking novel 22.9 kbp duplication of the chloroquine resistance associated gene *crt*, for which isolates are positive for both the CVMNK (chloroquine susceptible) and CVIET (chloroquine resistant) forms of the gene across multiple independent West African sub-populations. Whilst, the putative *crt* duplications need to be confirmed, the detection of those variants in isolates with a balanced number of CVMNK and CVIET haplotypes was robust to increasing the stringency on the number of supporting sequencing reads. It is unclear whether dual-carriage of these variants would allow expression of both or either forms of the *crt* transporter, though it is likely that this could allow individual parasites to benefit from chloroquine resistance with a reduced fitness cost. This finding is similar to previously identified alternatively spliced forms of *crt* in eastern Sudan which were hypothesised to facilitate ‘switching’ between chloroquine resistant and susceptible isoforms^20^. Further short ~29 bp and ~430 bp deletions identified here at low frequency in *crt* may reflect the specific deletions identified in that same study. Follow up studies, particularly with culture-adapted clinical isolates in which this duplication is present, are required to properly characterise *in vitro* phenotypes. We also present further characterisation of the promoter region duplication for the SP resistance associated gene *gch1*, previously identified in Malawi^5^. This additional analysis confirms the duplication is at near-fixation in Malawi, and highlights its presence across Central and East Africa, including notably high frequencies in Tanzania, Kenya, Guinea and the DRC. Further this genetic region has been shown to be under positive selection in Malawi using SNP-based metrics^5^.

Our results demonstrate that application of our pipeline can enhance the speed and capacity of high throughput structural variant discovery. However, this is not without limitations, especially as we rely upon validity of the underlying mapping, for which some regions (such as those which are highly variable or repetitive) are known to be difficult to characterise. To resolve this issue, we excluded known highly variable regions from our analysis, such as *var*, *rifin* and *stevor* genes and subtelomeric regions. However, in doing so we prevent discovery of true variants within these regions, including duplications in AT-rich loci^12^. In addition, all variants found were identified relative to the 3D7 reference strain, consistent with the approach taken in other studies^9,10^. Given that 3D7 is most likely an African strain, SVs within African isolates may be artificially under-represented due to those variants also being present within 3D7. Further, the discovery stage of our pipeline inherits the limitations of those tools, such as an inability to infer high quality inversions. This risk was limited by prioritising those variants that were identified by DELLY with support from an alternative discovery software (CNVNator or Control-FREEC).

The approach taken in this study, as with standard SNP discovery, requires single-genotype samples, preventing investigation of more complex isolates. By pre-screening for non-complex infections and also filtering on rates of predicted genotype, we were able to reduce false positive calls but also removed several highly likely variants that presented with a high prevalence of predicted heterozygous calls and potentially underestimate the total number of duplications. Notable candidate variants excluded from our high-quality dataset but supported by manual inspection of coverage depth or similar variants within the existing scientific literature include 102 putative deletions within the glycophorin A binding, invasion-critical gene *EBA175 (PF3D7_0731500*)^16^, the most prominent being a 424 bp deletion in 1,492 isolates (30.5% of isolates). We also identify a 586 bp deletion elevated in Africa (58.4% Africa, 12.5% America, 1.3% Asia) within *Rh2b*, a gene that plays a key role in erythrocyte invasion^15^. Similar deletions have previously been identified (and validated here) in the T996 *P. falciparum* line^21^ and in isolates from Senegal, where it is possibly associated with the utilisation of neuraminidase-sensitive invasion pathways^22^. Another variant is a 29 bp deletion in *rhopH2* (*PF3D7_0929400*), which has a reduced prevalence in Asian populations, and knockdowns of which have been shown to inhibit parasite growth within host erythrocytes^16^.

Our final count of 70,858 high quality specific variants assumes that each SV is distinct by their specific base-pair location. This means that we identify variants which arose from similar evolutionary events but may place insufficient emphasis on variants with a shared phenotypic impact. Previous studies collapsed analysis to a locus level, but risk overlooking complex structural variation within the same gene. This challenge was partially resolved via our windows-based approach, whereby variants are grouped due to their presence within a 1 kbp window.

Overall, our work presents a set of high-quality SVs, some population specific, which are likely to have functional consequences for drug resistance and erythrocyte invasion. An extended list of further structural variation requires both technological advances, such as low cost long read platforms with low error rates, as well as computational and algorithmic advances that assemble genomes to high accuracy and require less hands-on filtering. To facilitate further exploration of our full set of global structural variation by the wider scientific community we have developed a visualisation and analysis tool. This resource will assist much-needed genomic investigations into *P. falciparum*, potentially leading to biological insights for the development of disease control measures.

## Methods

### Sequence data

Illumina raw sequence data from more than 3,500 isolates in the Pf3k project were downloaded from the European Nucleotide Archive (see the project website, https://www.malariagen.net/projects/pf3k). The raw sequences were aligned to the *P. falciparum* 3D7 genome using bwa-mem software (settings: –c 100 –T 50)^23^, resulting in a mean coverage of 70.7-fold (Genic: 91.1-fold, Intergenic: 41.8-fold). SNPs and small indels (<15 bp) were called using SAMtools/BCFtools^24^ (default settings, version 1.6.1) and GATK software^25^ (settings: “-T UnifiedGenotyper -ploidy 1 -glm BOTH -allowPotentiallyMisencodedQuals 2”, version 3.4–46). Isolates bearing abnormal coverage less than 20-fold or greater than 300-fold coverage were excluded to reduce false positive rates. Similarly, isolate samples with complex infections were excluded by accessing multiplicity of infection (MOI) as rates of missing and heterozygous SNP calls (>20%) and using *estMOI* software^4^ (>20% genome with MOI > 1; established using the standard point of inflection approach^5^). Specific SVs were further filtered by their phasing, as predicted by DELLY, as a secondary control against cryptic mixed infections (see below for the stringent heterozygous genotype filtering). After quality control, our dataset included 2,855 isolates representing West Africa (n = 691), Central Africa (n = 344), East Africa (n = 464), South Asia (n = 43), Southeast Asia (n = 1,291), and South America (n = 22). The Pf3k Illumina data was supplemented by PacBio sequences (ERP009847) from 13 laboratory strains^12,26,27^, including 7G8 (Brazil), IT (Brazil), HB3 (Honduras), GA01 (Gabon), GN01 (Guinea), GB4 (Ghana), SN01 (Senegal), CD01 (Congo), KE01 (Kenya), SD01 (Sudan), DD2 (Indochina), KH01 (Cambodia), and KH02 (Cambodia).

### Structural variant discovery

Structural variants were predicted from short read alignments against the latest 3D7 reference assembly using DELLY (v0.7.3), which has been found to be robust across a range of organisms^13^. Smaller SVs, between 15 and 300 bp, were also identified with DELLY using the -i argument that utilises only soft-clipped read support. DELLY calling is considered more reliable for variants greater than 300 bp in length^13^, and our discussion of results is therefore predominantly of this group but recognises the presence of shorter variants of high frequency having strong support. The DELLY genotyping model (which assumes diploidy) was employed to call heterozygous SVs. Variants longer than 100,000 base pairs were excluded as a conservative filter for erroneous calls. Variants identified within 100 kbp of a chromosome end and in *var*, *rifin* and *stevor* genes were removed due to established difficulties in accurately mapping these regions^28^.

### Population-based SV Filtering and analysis

The SV-Pop (version 1.0) pipeline was utilised for post-discovery, population-based filtering of SVs and is publicly available from https://github.com/mattravenhall/SV-Pop. Population-wide filters were applied to exclude those variants with mean DELLY quality scores below 0.9, missingness >10%, an absence of paired read support, or homozygous reference calls frequency >10%. Variants were also removed if they displayed a heterozygous genotype frequency greater than 30%, as these suggest cryptic mixed infections not identified at the SNP calling stage. This filter was not applied for the candidate gene analysis. Regional hotspots were identified using sums of isolates with variants in 1 kbp sliding windows with a 500 bp step size. Ultimately our approach produced three lists of candidate SVs: (i) over 1 million putative variants identified by DELLY alone (raw dataset), (ii) our primary “high quality” set of 70,858 SVs following filtering by SV-Pop (high-quality dataset), and (iii) 92,313 “high quality relaxed” candidate SVs following relaxation of the SV-Pop parameters to include heterozygous duplications (high-quality relaxed dataset).

### Validation

The whole genomes from the 13 laboratory strains were used to validate any putative deletions and duplications detected by applying our discovery pipeline to the 2,855 isolates. Manual verification was performed for all SVs found in regions specifically mentioned in this manuscript (e.g. in drug resistance genes), and involved examination of per-base coverage plots and read pair alignments with the 2,855 Illumina samples. To further confirm high quality SVs, deletions and duplications were also identified using CNVNator (v0.3.2; bin size of 400 bp)^29^ and by Control-FREEC (v11.0; window size 100 bp, window step 50 bp, ploidy of 1)^11,30^ software. Concordance statistics between the variants detected on the DELLY pipeline and these alternative approaches were derived on a per variant basis in 1 kbp windows. Using either CNVNator and/or Control-FREEC we confirmed 97.4% for all high-quality variants detected using our DELLY pipeline, and there was 95.3% concordance for the subset of variants greater than 100 bp.

### Visualisation of variants

All SVs can be viewed using an online tool (http://genomics.lshtm.ac.uk/PfGlobalSV/) (developed using SV-pop platform software^31^), where the full list of isolates (n = 2,855) with ENA codes are presented. This tool and our analysis compare variant frequencies between populations. Specifically, multi-population F_ST_ statistics were calculated between continent (Africa, Asia, South America) and region-based sub-populations (West Africa, Central Africa, East Africa, South Asia, Southeast Asia, South America) for both windows and variants using Nei’s method^32^.

### Calculation of *crt* haplotype abundance

To determine the variability of *crt* in duplication positive isolates, we conducted strict match read counts with high quality pre-alignment reads for five specific haplotypes. Haplotype sequences were 25 base pairs long, and included CVMVK (TGTATGTGTAATGAATAAAATTTTT), CVIET (TGTATGTGTAATTGAAACAATTTTT), CVIDT (TGTATGTGTAATTGATACAATTTTT), CVMET (TGTATGTGTAATGGAAACAATTTTT), and CVMNT (TGTATGTGTAATGAATACAATTTTT). Only CVIET and CVMNK haplotypes were observed in West Africa, and the proportion of CVIET reads for each isolate was calculated. These analyses were performed on the high-quality relaxed dataset.

## Supplementary information

Supplementary info

## Data Availability

For the primary short read data set, public accession numbers for the raw sequence data analysed are contained in SRA studies ERP000190 and ERP000199, as well as being accessible from the Pf3k project website (https://www.malariagen.net/projects/pf3k). Raw PacBio sequence data is available from the European Nucleotide Archive (ERP009847).
