# Supplementary material for: An analysis of large structural variation in global Plasmodium falciparum isolates identifies a novel duplication of the chloroquine resistance associated gene
Source: Sci Rep. 2019 Jun 4;9:8287. doi: 10.1038/s41598-019-44599-0 (PMC6547842; doi:10.1038/s41598-019-44599-0)
Supplement: Supplementary file 1 — Supplementary info [file 41598_2019_44599_MOESM1_ESM.pdf]

An analysis of large structural variation in global *Plasmodium falciparum* isolates identifies a novel duplication of the chloroquine resistance associated gene

Matt Ravenhall[1], Ernest Diez Benavente[1], Colin J. Sutherland[2], David A. Baker[1], Susana Campino[1,*], Taane G. Clark[1,3,*]

1. Department of Pathogen Molecular Biology, London School of Hygiene and Tropical Medicine, London WC1E 7HT, UK
2. Department of Immunology and Infection, London School of Hygiene and Tropical Medicine, London WC1E 7HT, UK
3. Department of Infectious Disease Epidemiology, Faculty of Epidemiology and Population Health, London School of Hygiene and Tropical Medicine, London WC1E 7HT, UK

*Joint last authors

Supplementary figure 1

Density plot* showing the proportion of CVIET haplotype reads (from the total of CVIET and CVMNK reads observed) amongst West African isolates with and without *crt* duplications.

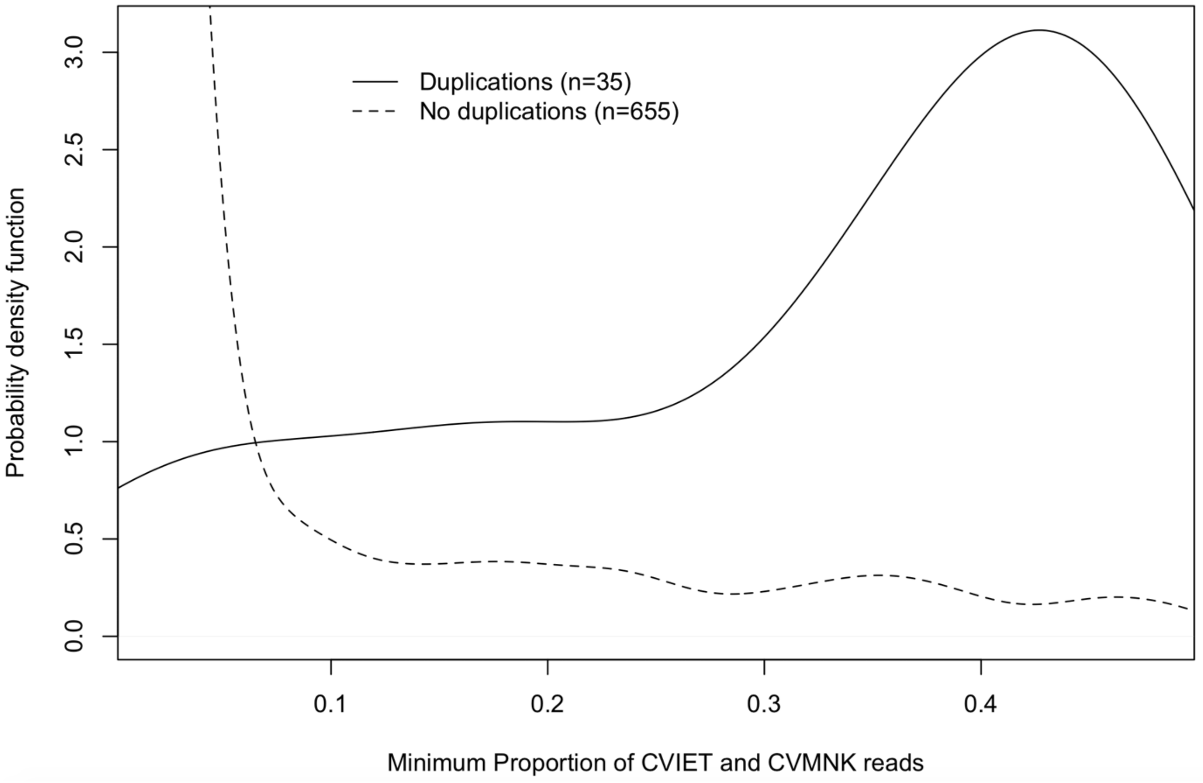

*Probability density function for the kernel density estimation.

Supplementary table 1

Summary of the set of isolates analysed (n=2,855)

Region/Country	N	%
West Africa	*691*	*24.2*
Burkina Faso	45	*1.6*
The Gambia	65	*2.3*
Ghana	413	*14.5*
Guinea	109	*3.8*
Mali	54	*1.9*
Nigeria	5	*0.2*
Central Africa	*344*	*12.0*
Cameroon	127	*4.4*
Demo. Rep. Congo	217	*7.6*
East Africa	*464*	*16.3*
Kenya	38	*1.3*
Madagascar	18	*0.6*
Malawi	347	*12.2*
Tanzania	61	*2.1*
Bangladesh (South Asia)	*43*	*1.5*
Southeast Asia	*1435*	*50.3*
Cambodia	548	*19.2*
Laos	104	*3.6*
Myanmar	123	*4.3*
Thailand	308	*10.8*
Vietnam	183	*6.4*
Papua New Guinea	25	*0.9*
South America	*22*	*0.8*
Colombia	13	*0.5*
Peru	9	*0.3*
Total	2855	*100*

Supplementary table 2

Genes with common variants (>1% frequency) greater than 300bp in length in the pre-filtered data

Gene	Gene Name	Type	Freq.	West Africa	Central Africa	East Africa	South Asia	SEA	South America	Regional F_{ST}
PF3D7_1038400	*Pf11-1*	DUP	2778	0.973	0.988	0.978	0.953	0.968	0.955	0.005
PF3D7_1115300	*FP2B*	DUP	1750	0.389	0.529	0.435	0.628	0.826	0.182	0.161
PF3D7_1115400	*FP3*	DUP	1750	0.389	0.529	0.435	0.628	0.826	0.182	0.161
PF3D7_1115500	*ApiAP2*	DUP	1750	0.389	0.529	0.435	0.628	0.826	0.182	0.161
PF3D7_1115600	*CYP19B*	DUP	1750	0.389	0.529	0.435	0.628	0.826	0.182	0.161
PF3D7_1115700	*FP2A*	DUP	1746	0.385	0.529	0.435	0.628	0.825	0.182	0.161
PF3D7_1442600	*TREP*	DUP	1744	0.499	0.791	0.718	0.651	0.590	0.182	0.159
PF3D7_0526600	-	DUP	1468	0.305	0.701	0.599	0.628	0.548	0.136	0.159
PF3D7_0504700	*CEP120*	DUP	1035	0.440	0.328	0.353	0.302	0.332	0.545	0.030
PF3D7_1417900	-	DEL	1021	0.420	0.328	0.375	0.442	0.319	0.591	0.034
PF3D7_1225300	-	DEL	922	0.350	0.235	0.287	0.372	0.336	0.727	0.108
PF3D7_1035900	*M566*	DUP	827	0.343	0.276	0.334	0.233	0.256	0.000	0.072
PF3D7_1035700	*DBLMSP*	DUP	773	0.320	0.244	0.310	0.233	0.243	0.000	0.065
PF3D7_1035800	*M712*	DUP	773	0.320	0.244	0.310	0.233	0.243	0.000	0.065
PF3D7_1036300	*DBLMSP2*	DUP	771	0.320	0.244	0.308	0.233	0.242	0.000	0.064
PF3D7_1036000	*MSP11*	DUP	770	0.320	0.244	0.308	0.233	0.242	0.000	0.064
PF3D7_1038400	*Pf11-1*	DEL	630	0.194	0.140	0.134	0.163	0.288	0.318	0.031
PF3D7_1318300	-	DEL	578	0.253	0.052	0.127	0.047	0.246	0.318	0.076
PF3D7_1241800	*DBP9*	DUP	554	0.136	0.160	0.166	0.279	0.239	0.318	0.026
PF3D7_0113100	*SURF1.1*	DUP	407	0.091	0.131	0.103	0.140	0.187	0.182	0.011
PF3D7_0113200	-	DUP	407	0.090	0.131	0.103	0.140	0.187	0.182	0.011
PF3D7_0113300	-	DUP	406	0.090	0.131	0.101	0.140	0.187	0.182	0.011
PF3D7_0113400	-	DUP	404	0.090	0.131	0.101	0.140	0.186	0.182	0.011
PF3D7_1224000	*GCH1*	DUP	307	0.032	0.035	0.009	0.209	0.201	0.000	0.105
PF3D7_0504700	*CEP120*	DEL	243	0.107	0.055	0.034	0.023	0.101	0.136	0.024
PF3D7_1223800	*YHM2*	DUP	226	0.019	0.020	0.002	0.209	0.152	0.000	0.108
PF3D7_1223700	*VIT*	DUP	225	0.017	0.020	0.002	0.209	0.152	0.000	0.109
PF3D7_1223900	-	DUP	225	0.017	0.020	0.000	0.209	0.153	0.000	0.111
PF3D7_1362700	-	DUP	212	0.051	0.081	0.091	0.070	0.081	0.000	0.016
PF3D7_1024900	-	DEL	206	0.000	0.000	0.000	0.000	0.160	0.000	0.137
PF3D7_0220000	*LSA3*	DEL	202	0.007	0.009	0.017	0.070	0.141	0.045	0.048
PF3D7_0417000	-	DEL	198	0.069	0.055	0.097	0.070	0.064	0.000	0.016
PF3D7_1035800	*M712*	DEL	198	0.094	0.032	0.037	0.023	0.071	0.545	0.297
PF3D7_1352600	-	DEL	197	0.052	0.093	0.099	0.023	0.064	0.000	0.024
PF3D7_0523000	*MDR1*	DUP	194	0.001	0.000	0.000	0.000	0.149	0.000	0.126
PF3D7_0523100	-	DUP	171	0.001	0.000	0.000	0.000	0.132	0.000	0.111
PF3D7_0511500	-	DEL	168	0.000	0.000	0.011	0.023	0.125	0.000	0.078

PF3D7_1224100	-	DUP	161	0.003	0.020	0.006	0.047	0.114	0.000	0.052
PF3D7_0220000	*LSA3*	DUP	159	0.058	0.119	0.024	0.070	0.048	0.091	0.015
PF3D7_1126900	*SNRPF*	DUP	152	0.098	0.145	0.056	0.000	0.000	0.364	0.158
PF3D7_1407900	*PMI*	DUP	152	0.003	0.003	0.000	0.000	0.115	0.000	0.091
PF3D7_1408000	*PMII*	DUP	152	0.003	0.003	0.000	0.000	0.115	0.000	0.091
PF3D7_1127000	-	DUP	151	0.098	0.145	0.054	0.000	0.000	0.364	0.158
PF3D7_1408100	*HAP*	DUP	151	0.003	0.003	0.000	0.000	0.115	0.000	0.091
PF3D7_1146700	-	DEL	143	0.067	0.061	0.052	0.000	0.040	0.045	0.011
PF3D7_1467600	-	DUP	140	0.072	0.084	0.084	0.000	0.016	0.045	0.023
PF3D7_1012800	-	DEL	138	0.087	0.017	0.026	0.023	0.044	0.091	0.020
PF3D7_0523200	-	DUP	129	0.000	0.000	0.002	0.000	0.099	0.000	0.082
PF3D7_0522900	-	DUP	126	0.001	0.000	0.006	0.000	0.095	0.000	0.073
PF3D7_0503000	-	DEL	104	0.048	0.032	0.078	0.047	0.017	0.000	0.017
PF3D7_1321100	-	DUP	101	0.014	0.026	0.043	0.140	0.043	0.000	0.049
PF3D7_1237900	-	DEL	98	0.001	0.003	0.002	0.047	0.072	0.000	0.039
PF3D7_1476300	-	DEL	98	0.036	0.029	0.019	0.070	0.040	0.000	0.015
PF3D7_1228400	-	DEL	96	0.055	0.026	0.032	0.000	0.025	0.091	0.022
PF3D7_1308700	-	DEL	89	0.006	0.003	0.004	0.000	0.064	0.000	0.042
PF3D7_1316600	*CCT*	DUP	88	0.049	0.044	0.022	0.047	0.021	0.000	0.011
PF3D7_1434200	*CAM*	DEL	87	0.032	0.020	0.045	0.023	0.027	0.045	0.003
PF3D7_1360500	*GCbeta*	DEL	74	0.023	0.000	0.024	0.000	0.036	0.045	0.014
PF3D7_1130500	*COG2*	DEL	60	0.022	0.006	0.028	0.000	0.023	0.000	0.010
PF3D7_1127000	-	DEL	58	0.017	0.108	0.015	0.000	0.000	0.091	0.052
PF3D7_1324000	-	DEL	58	0.001	0.006	0.004	0.000	0.041	0.000	0.025
PF3D7_1206300	-	DUP	57	0.027	0.012	0.030	0.000	0.015	0.000	0.010
PF3D7_1412400	-	DEL	56	0.020	0.003	0.004	0.070	0.028	0.000	0.029
PF3D7_1467600	-	DEL	52	0.013	0.012	0.024	0.000	0.022	0.000	0.008
PF3D7_0310200	-	DUP	51	0.007	0.017	0.034	0.023	0.018	0.000	0.007
PF3D7_1107300	*PAIP1*	DUP	51	0.013	0.000	0.034	0.023	0.019	0.000	0.010
PF3D7_1223000	-	DEL	50	0.004	0.003	0.099	0.000	0.000	0.000	0.076
PF3D7_0802700	-	DEL	49	0.032	0.020	0.030	0.023	0.003	0.045	0.007
PF3D7_0933200	-	DEL	49	0.012	0.000	0.015	0.023	0.026	0.000	0.008
PF3D7_1036400	*LSA1*	DUP	49	0.006	0.006	0.009	0.000	0.030	0.000	0.012
PF3D7_0619000	-	DEL	48	0.013	0.003	0.017	0.000	0.023	0.000	0.009
PF3D7_1442600	*TREP*	DEL	48	0.043	0.020	0.011	0.000	0.005	0.000	0.017
PF3D7_0406500	-	DUP	46	0.009	0.009	0.039	0.000	0.015	0.000	0.015
PF3D7_0511500	-	DUP	46	0.010	0.006	0.032	0.023	0.015	0.045	0.008
PF3D7_1318400	*SMC2*	DEL	45	0.014	0.015	0.015	0.023	0.017	0.000	0.003
PF3D7_1337500	-	DEL	45	0.016	0.015	0.015	0.000	0.017	0.000	0.005
PF3D7_0312000	-	DEL	44	0.010	0.003	0.004	0.000	0.026	0.000	0.012
PF3D7_0826100	*HEUL*	DUP	44	0.007	0.003	0.037	0.023	0.015	0.000	0.012
PF3D7_0829500	-	DUP	44	0.009	0.000	0.037	0.023	0.015	0.000	0.012
PF3D7_0508900	-	DUP	43	0.016	0.006	0.037	0.000	0.010	0.000	0.014

PF3D7_0604500	-	DUP	42	0.009	0.000	0.028	0.023	0.017	0.000	0.009
PF3D7_0709300	*CG2*	DUP	42	0.052	0.000	0.011	0.000	0.001	0.000	0.034
PF3D7_0925200	*RRP8*	DEL	38	0.013	0.000	0.002	0.000	0.022	0.000	0.012
PF3D7_0709000	*CRT*	DUP	37	0.051	0.000	0.000	0.000	0.002	0.000	0.041
PF3D7_0709100	-	DUP	37	0.051	0.000	0.002	0.000	0.001	0.000	0.040
PF3D7_1035400	*MSP3*	DUP	37	0.013	0.015	0.004	0.000	0.016	0.000	0.006
PF3D7_0310200	-	DEL	36	0.019	0.012	0.009	0.023	0.010	0.045	0.008
PF3D7_0709050	-	DUP	36	0.051	0.000	0.000	0.000	0.001	0.000	0.042
PF3D7_0709200	*GLP3*	DUP	36	0.051	0.000	0.000	0.000	0.001	0.000	0.042
PF3D7_1021900	-	DUP	36	0.006	0.000	0.030	0.023	0.013	0.000	0.011
PF3D7_1235300	*NOT4*	DUP	36	0.010	0.000	0.032	0.023	0.010	0.000	0.011
PF3D7_0421700	-	DEL	35	0.017	0.000	0.015	0.000	0.012	0.000	0.008
PF3D7_1324400	*PRELID*	DEL	35	0.013	0.000	0.015	0.000	0.015	0.000	0.007
PF3D7_0708900	*SCO1*	DUP	35	0.051	0.000	0.000	0.000	0.000	0.000	0.043
PF3D7_1237900	-	DUP	35	0.014	0.006	0.015	0.023	0.012	0.000	0.005
PF3D7_1417200	-	DUP	35	0.007	0.003	0.028	0.023	0.012	0.000	0.009
PF3D7_0104300	*UBP1*	DUP	34	0.006	0.000	0.026	0.023	0.013	0.000	0.010
PF3D7_1249200	-	DEL	33	0.025	0.006	0.024	0.000	0.002	0.000	0.012
PF3D7_0207900	*SERA2*	DUP	33	0.019	0.023	0.000	0.000	0.009	0.000	0.011
PF3D7_0519500	*CCR4*	DUP	33	0.006	0.000	0.032	0.023	0.010	0.000	0.012
PF3D7_0704100	-	DUP	33	0.004	0.000	0.028	0.023	0.012	0.000	0.011
PF3D7_0202400	*PTEF*	DUP	32	0.006	0.000	0.022	0.023	0.013	0.000	0.008
PF3D7_0801900	*LSD2*	DUP	32	0.006	0.000	0.026	0.023	0.012	0.000	0.010
PF3D7_1423700	-	DUP	32	0.013	0.000	0.024	0.000	0.009	0.000	0.010
PF3D7_0613900	*myoE*	DUP	31	0.009	0.023	0.011	0.000	0.009	0.000	0.007
PF3D7_0526600	-	DEL	30	0.010	0.026	0.002	0.000	0.010	0.000	0.010
PF3D7_1036400	*LSA1*	DEL	30	0.007	0.006	0.004	0.000	0.016	0.000	0.005
PF3D7_1351400	-	DEL	30	0.010	0.003	0.011	0.023	0.012	0.000	0.006
PF3D7_1351500	-	DEL	30	0.010	0.003	0.011	0.023	0.012	0.000	0.006
PF3D7_0207800	*SERA3*	DUP	30	0.019	0.009	0.002	0.000	0.010	0.000	0.007
PF3D7_0627900	*POP4*	DUP	30	0.000	0.000	0.000	0.000	0.023	0.000	0.019
PF3D7_0704300	-	DUP	30	0.007	0.000	0.030	0.000	0.009	0.000	0.015
PF3D7_1017600	-	DUP	30	0.006	0.000	0.022	0.023	0.012	0.000	0.009
PF3D7_1409100	-	DUP	30	0.006	0.000	0.028	0.000	0.010	0.000	0.014
PF3D7_0615400	-	DUP	29	0.006	0.000	0.028	0.000	0.009	0.000	0.014
PF3D7_0903300	-	DUP	29	0.009	0.000	0.028	0.000	0.008	0.000	0.013
PF3D7_1021700	-	DUP	29	0.003	0.000	0.028	0.023	0.010	0.000	0.012

SEA = Southeast Asia; DUP = duplication; DEL = deletion

Supplementary table 3

Most frequent identical high-quality variants (>300bp) in the global population

Feature	Gene name	SV Type	Freq.	West Africa	Central Africa	East Africa	South Asia	SEA	South America	Regional F_{ST}
PF3D7_1417900	-	DEL	1021	0.420	0.328	0.375	0.442	0.319	0.591	0.034
PF3D7_1225300	-	DEL	922	0.350	0.235	0.287	0.372	0.336	0.727	0.108
PF3D7_1318300	-	DEL	578	0.253	0.052	0.127	0.047	0.246	0.318	0.076
PF3D7_0504700	*CEP120*	DEL	242	0.107	0.052	0.034	0.023	0.101	0.136	0.025
PF3D7_1024900	-	DEL	206	0.000	0.000	0.000	0.000	0.160	0.000	0.137
PF3D7_0220000	*LSA3*	DEL	202	0.007	0.009	0.017	0.070	0.141	0.045	0.048
PF3D7_0417000	-	DEL	198	0.069	0.055	0.097	0.070	0.064	0.000	0.016
PF3D7_1352600	-	DEL	197	0.052	0.093	0.099	0.023	0.064	0.000	0.024
PF3D7_1035800	*M712*	DEL	195	0.093	0.032	0.034	0.023	0.071	0.500	0.261
PF3D7_0504700	*CEP120*	DUP	144	0.084	0.035	0.047	0.023	0.038	0.091	0.013
PF3D7_1146700	-	DEL	143	0.067	0.061	0.052	0.000	0.040	0.045	0.011
PF3D7_1012800	-	DEL	138	0.087	0.017	0.026	0.023	0.044	0.091	0.020
PF3D7_0503000	-	DEL	104	0.048	0.032	0.078	0.047	0.017	0.000	0.017
PF3D7_1237900	-	DEL	98	0.001	0.003	0.002	0.047	0.072	0.000	0.039
PF3D7_1476300	-	DEL	98	0.036	0.029	0.019	0.070	0.040	0.000	0.015
PF3D7_1228400	-	DEL	96	0.055	0.026	0.032	0.000	0.025	0.091	0.022
PF3D7_1308700	-	DEL	89	0.006	0.003	0.004	0.000	0.064	0.000	0.042
PF3D7_1360500	*GCbeta*	DEL	73	0.023	0.000	0.024	0.000	0.035	0.045	0.013
PF3D7_1130500	*COG2*	DEL	60	0.022	0.006	0.028	0.000	0.023	0.000	0.010
PF3D7_1127000	-	DEL	58	0.017	0.108	0.015	0.000	0.000	0.091	0.052
PF3D7_1324000	-	DEL	58	0.001	0.006	0.004	0.000	0.041	0.000	0.025
PF3D7_1412400	-	DEL	56	0.020	0.003	0.004	0.070	0.028	0.000	0.029
PF3D7_1467600	-	DEL	52	0.013	0.012	0.024	0.000	0.022	0.000	0.008
PF3D7_1223000	-	DEL	50	0.004	0.003	0.099	0.000	0.000	0.000	0.076
PF3D7_0802700	-	DEL	49	0.032	0.020	0.030	0.023	0.003	0.045	0.007
PF3D7_0933200	-	DEL	49	0.012	0.000	0.015	0.023	0.026	0.000	0.008
PF3D7_1442600	*TREP*	DEL	48	0.043	0.020	0.011	0.000	0.005	0.000	0.017
PF3D7_1337500	-	DEL	45	0.016	0.015	0.015	0.000	0.017	0.000	0.005
PF3D7_0312000	-	DEL	43	0.009	0.003	0.004	0.000	0.026	0.000	0.012
PF3D7_1318400	*SMC2*	DEL	41	0.013	0.012	0.015	0.023	0.015	0.000	0.004
PF3D7_0925200	*RRP8*	DEL	38	0.013	0.000	0.002	0.000	0.022	0.000	0.012
PF3D7_0310200	-	DEL	36	0.019	0.012	0.009	0.023	0.010	0.045	0.008
PF3D7_0421700	-	DEL	35	0.017	0.000	0.015	0.000	0.012	0.000	0.008
PF3D7_1324400	*PRELID*	DEL	35	0.013	0.000	0.015	0.000	0.015	0.000	0.007
PF3D7_0526600	-	DEL	30	0.010	0.026	0.002	0.000	0.010	0.000	0.010
PF3D7_1036400	*LSA1*	DEL	30	0.007	0.006	0.004	0.000	0.016	0.000	0.005
PF3D7_1351400	-	DEL	30	0.010	0.003	0.011	0.023	0.012	0.000	0.006
PF3D7_1351500	-	DEL	30	0.010	0.003	0.011	0.023	0.012	0.000	0.006
PF3D7_0819700	-	DEL	28	0.023	0.006	0.004	0.000	0.006	0.000	0.009

PF3D7_0106400	-	DEL	26	0.014	0.003	0.006	0.023	0.009	0.000	0.006
PF3D7_0208300	-	DEL	26	0.006	0.000	0.004	0.000	0.015	0.000	0.007
PF3D7_1321100	-	DEL	25	0.007	0.023	0.015	0.000	0.004	0.000	0.009
PF3D7_0528400	*DHHC7*	DEL	24	0.007	0.003	0.034	0.000	0.002	0.000	0.019
PF3D7_0807000	-	DEL	22	0.009	0.006	0.019	0.000	0.004	0.000	0.007
PF3D7_1238500	-	DEL	22	0.001	0.000	0.000	0.000	0.016	0.000	0.012
PF3D7_0623900	-	DEL	20	0.020	0.009	0.002	0.023	0.000	0.045	0.014
PF3D7_1410300	-	DEL	20	0.001	0.012	0.002	0.000	0.011	0.000	0.006
PF3D7_1406000	*RBM8A*	DEL	18	0.006	0.003	0.006	0.000	0.008	0.000	0.002
PF3D7_0829800	-	DEL	17	0.006	0.006	0.009	0.000	0.005	0.000	0.003
PF3D7_0220000	*LSA3*	DUP	17	0.000	0.000	0.000	0.000	0.013	0.000	0.011
PF3D7_1327000	-	DEL	16	0.012	0.003	0.000	0.000	0.005	0.000	0.006
PF3D7_0406500	-	DEL	15	0.003	0.006	0.000	0.000	0.008	0.045	0.024
PF3D7_1224000	*GCH1*	DUP	15	0.001	0.017	0.000	0.047	0.005	0.000	0.025
PF3D7_0814100	-	DEL	14	0.006	0.003	0.011	0.000	0.002	0.045	0.022
PF3D7_1216200	-	DEL	14	0.001	0.000	0.006	0.000	0.008	0.000	0.004
PF3D7_1335300	*RH2b*	DEL	14	0.009	0.003	0.013	0.000	0.001	0.000	0.006
PF3D7_1362700	-	DEL	14	0.013	0.003	0.002	0.000	0.002	0.000	0.006
PF3D7_1453200	-	DEL	14	0.001	0.000	0.002	0.023	0.009	0.000	0.012
PF3D7_1463900	-	DEL	14	0.010	0.000	0.006	0.000	0.002	0.045	0.024
PF3D7_0629300	*PL*	DEL	13	0.009	0.009	0.004	0.000	0.002	0.000	0.004
PF3D7_0217900	-	DEL	12	0.009	0.006	0.004	0.000	0.002	0.000	0.003
PF3D7_0508900	-	DEL	12	0.004	0.009	0.006	0.000	0.002	0.000	0.003
PF3D7_0528500	*CPalpha*	DEL	12	0.003	0.000	0.011	0.000	0.004	0.000	0.005
PF3D7_1116000	*RON4*	DEL	12	0.000	0.006	0.000	0.000	0.008	0.000	0.005
PF3D7_0423900	-	DEL	11	0.010	0.000	0.004	0.047	0.000	0.000	0.028
PF3D7_0619800	-	DEL	11	0.003	0.003	0.000	0.000	0.006	0.000	0.003
PF3D7_0826400	-	DEL	11	0.001	0.003	0.002	0.000	0.006	0.000	0.002
PF3D7_1114700	*CLK3*	DEL	11	0.000	0.006	0.002	0.000	0.006	0.000	0.003
PF3D7_0405300	*LISP2*	DEL	10	0.000	0.000	0.000	0.000	0.008	0.000	0.007
PF3D7_0419900	-	DEL	10	0.004	0.000	0.002	0.023	0.004	0.000	0.012
PF3D7_1009500	-	DEL	10	0.000	0.000	0.000	0.000	0.008	0.000	0.007
PF3D7_1012400	*HGPRT*	DEL	10	0.001	0.003	0.009	0.023	0.002	0.000	0.010
PF3D7_1202100	-	DEL	10	0.001	0.000	0.004	0.000	0.005	0.000	0.003
PF3D7_1461800	-	DEL	10	0.000	0.012	0.004	0.000	0.003	0.000	0.006
PF3D7_0210100	-	DEL	9	0.006	0.003	0.000	0.000	0.003	0.000	0.003
PF3D7_1035900	*M566*	DEL	9	0.003	0.003	0.004	0.000	0.001	0.136	0.104
PF3D7_1211300	*MCM8*	DEL	9	0.007	0.000	0.004	0.000	0.002	0.000	0.003
PF3D7_0523000	*MDR1*	DUP	9	0.000	0.000	0.000	0.000	0.007	0.000	0.006
PF3D7_0523100	-	DUP	9	0.000	0.000	0.000	0.000	0.007	0.000	0.006
PF3D7_1115300	*FP2B*	DUP	9	0.000	0.006	0.004	0.000	0.004	0.000	0.003
PF3D7_1115400	*FP3*	DUP	9	0.000	0.006	0.004	0.000	0.004	0.000	0.003
PF3D7_1115500	*ApiAP2*	DUP	9	0.000	0.006	0.004	0.000	0.004	0.000	0.003

PF3D7_1115600	*CYP19B*	DUP	9	0.000	0.006	0.004	0.000	0.004	0.000	0.003
PF3D7_1115700	*FP2A*	DUP	9	0.000	0.006	0.004	0.000	0.004	0.000	0.003
PF3D7_1442600	*TREP*	DUP	9	0.006	0.003	0.004	0.000	0.002	0.000	0.002
PF3D7_0113800	-	DEL	8	0.003	0.003	0.000	0.000	0.004	0.000	0.002
PF3D7_0514200	-	DEL	8	0.001	0.003	0.004	0.023	0.002	0.000	0.012
PF3D7_1108100	-	DEL	8	0.007	0.006	0.002	0.000	0.000	0.000	0.003
PF3D7_1120000	-	DEL	8	0.006	0.003	0.002	0.000	0.002	0.000	0.002
PF3D7_1223700	*VIT*	DUP	8	0.000	0.003	0.000	0.047	0.004	0.000	0.033
PF3D7_1223800	*YHM2*	DUP	8	0.000	0.003	0.000	0.047	0.004	0.000	0.033
PF3D7_1223900	-	DUP	8	0.000	0.003	0.000	0.047	0.004	0.000	0.033

SEA = Southeast Asia; DUP = duplication; DEL = deletion

Supplementary table 4

Most frequent high-quality variants in the global population

Chr.	Start	Length	Freq.	West Africa	Central Africa	East Africa	South Asia	SEA	South America	Regional F_{ST}	Feature *PF3D7_*	Model
10	1186067	29	0.862	0.776	0.836	0.862	0.547	0.767	0.708	0.057		DEL
7	664587	24	0.839	0.745	0.792	0.811	0.660	0.759	0.917	0.035		DEL
11	1648740	31	0.780	0.603	0.592	0.628	0.679	0.833	0.458	0.054		DEL
14	353088	37	0.778	0.604	0.658	0.695	0.623	0.786	0.792	0.025		DEL
4	388088	28	0.766	0.565	0.689	0.726	0.698	0.763	0.667	0.018	*0407800*	DEL
8	552685	43	0.749	0.302	0.664	0.870	0.698	0.819	0.792	0.164		DEL
14	1380817	73	0.744	0.564	0.603	0.611	0.604	0.789	0.417	0.049	*1434500*	DEL
8	1307219	41	0.725	0.755	0.828	0.739	0.472	0.557	0.667	0.067	*0830700*	DEL
9	1420945	64	0.691	0.588	0.200	0.335	0.453	0.865	0.625	0.184	*0935900*	DEL
14	206374	28	0.675	0.428	0.853	0.757	0.585	0.616	0.208	0.183	*1405900*	DEL
4	409225	73	0.664	0.427	0.808	0.634	0.642	0.645	0.208	0.151	*0408500*	DEL
9	350069	31	0.662	0.539	0.603	0.669	0.453	0.624	0.625	0.021		DEL
3	838759	35	0.639	0.568	0.561	0.689	0.264	0.578	0.375	0.081		DEL
8	1248470	46	0.636	0.680	0.828	0.763	0.396	0.421	0.417	0.131	*0829000*	DEL
5	765224	19	0.635	0.627	0.661	0.685	0.509	0.508	0.458	0.031	*0518500*	DEL
7	687491	44	0.620	0.473	0.525	0.510	0.566	0.652	0.000	0.179		DEL
9	1180498	29	0.598	0.678	0.744	0.714	0.358	0.377	0.917	0.174	*0929400*	DEL
4	539258	29	0.594	0.558	0.592	0.626	0.377	0.499	0.750	0.053		DEL
13	465164	51	0.589	0.408	0.392	0.496	0.358	0.663	0.500	0.041	*1310600*	DEL
13	2079948	35	0.579	0.446	0.456	0.675	0.377	0.550	0.375	0.044		DEL
14	950743	28	0.572	0.413	0.794	0.695	0.604	0.457	0.208	0.151	*1423500*	DEL
13	2312787	25	0.559	0.247	0.250	0.329	0.679	0.772	0.000	0.301	*1358200*	DEL
14	1806644	25	0.559	0.401	0.439	0.508	0.547	0.583	0.542	0.016	*1444100*	DEL
9	686923	22	0.558	0.576	0.333	0.498	0.377	0.533	0.375	0.033	*0916400*	DEL
13	2696633	31	0.554	0.351	0.625	0.607	0.547	0.530	0.042	0.167	*1367700*	DEL
6	983169	27	0.545	0.379	0.581	0.660	0.283	0.497	0.167	0.118		DEL
14	646941	19	0.542	0.392	0.467	0.477	0.472	0.563	0.458	0.010	*1416100*	DEL
12	1203277	79	0.525	0.411	0.350	0.601	0.302	0.515	0.417	0.041		DEL
12	1443406	19	0.522	0.442	0.494	0.574	0.302	0.465	0.458	0.026	*1234600*	DEL
5	216506	67	0.520	0.553	0.592	0.574	0.434	0.378	0.458	0.025	*0505000*	DEL
6	1151260	64	0.520	0.163	0.469	0.387	0.717	0.661	0.167	0.190	*0628100*	DEL
5	1193026	28	0.517	0.297	0.383	0.305	0.453	0.644	0.208	0.083	*0529200*	DEL
13	1602337	37	0.514	0.389	0.458	0.469	0.585	0.507	0.625	0.025	*1340000*	DEL
14	1512540	29	0.514	0.438	0.389	0.449	0.208	0.522	0.500	0.044		DEL
12	165906	37	0.506	0.275	0.281	0.348	0.491	0.647	0.000	0.178		DEL
4	491496	67	0.505	0.247	0.575	0.521	0.566	0.524	0.167	0.108	*0410900*	DEL
5	1192722	39	0.505	0.125	0.200	0.095	0.604	0.821	0.042	0.396		DEL
11	737028	37	0.490	0.171	0.150	0.158	0.509	0.763	0.042	0.305	*1119500*	DEL
11	1085813	25	0.489	0.327	0.311	0.344	0.264	0.583	0.375	0.045		DEL

9	243657	42	0.487	0.180	0.217	0.154	0.472	0.738	0.000	0.284		DEL
8	835628	73	0.482	0.367	0.461	0.506	0.434	0.454	0.292	0.020		DEL
12	754958	33	0.482	0.322	0.333	0.267	0.264	0.591	0.375	0.053		DEL
10	1448825	29	0.475	0.340	0.350	0.506	0.302	0.482	0.583	0.043	*1036800*	DEL
11	1735609	25	0.469	0.466	0.428	0.438	0.340	0.407	0.625	0.031	*1143400*	DEL
14	1442706	28	0.463	0.226	0.433	0.553	0.547	0.476	0.208	0.082	*1435600*	DEL
5	588430	25	0.463	0.286	0.283	0.288	0.491	0.576	0.042	0.133	*0513900*	DEL
8	1274121	28	0.456	0.320	0.414	0.352	0.377	0.494	0.167	0.044	*0829800*	DEL
11	994250	55	0.456	0.320	0.561	0.498	0.528	0.406	0.042	0.130	*1125300*	DEL
12	1858154	22	0.455	0.283	0.372	0.352	0.302	0.515	0.625	0.062		DEL
9	1136899	25	0.453	0.276	0.286	0.379	0.321	0.533	0.292	0.035		DEL
10	222220	21	0.453	0.293	0.375	0.309	0.434	0.522	0.250	0.037	*1004800*	DEL
12	1943271	45	0.453	0.276	0.269	0.185	0.396	0.590	0.750	0.163		DEL
3	506737	20	0.449	0.390	0.392	0.407	0.302	0.431	0.458	0.010	*0311800*	DEL
9	829982	37	0.448	0.218	0.508	0.393	0.415	0.490	0.208	0.061	*0920200*	DEL
13	1465509	46	0.448	0.271	0.322	0.463	0.377	0.477	0.833	0.135	*1335900*	DEL
6	548419	25	0.445	0.103	0.158	0.140	0.472	0.714	0.042	0.293	*0613500*	DEL
2	724030	49	0.442	0.413	0.561	0.595	0.245	0.309	0.083	0.137	*0217600*	DEL
7	604035	22	0.441	0.271	0.319	0.374	0.321	0.507	0.208	0.039		DEL
8	1192129	46	0.439	0.317	0.467	0.463	0.264	0.417	0.125	0.066	*0827600*	DEL
4	249735	25	0.438	0.709	0.753	0.644	0.189	0.081	0.625	0.278	*0404600*	DEL
11	255648	29	0.437	0.332	0.594	0.502	0.321	0.358	0.250	0.058		DEL
7	175334	16	0.435	0.436	0.525	0.508	0.321	0.316	0.292	0.037	*0704000*	DEL
7	357252	25	0.435	0.156	0.258	0.113	0.283	0.661	0.000	0.234		DEL
12	1583456	57	0.435	0.122	0.122	0.193	0.396	0.679	0.000	0.269		DEL
7	144688	41	0.424	0.401	0.556	0.506	0.189	0.304	0.583	0.082	*0703600*	DEL
8	479558	28	0.416	0.329	0.308	0.374	0.528	0.427	0.000	0.121		DEL
2	504157	25	0.415	0.314	0.608	0.547	0.377	0.305	0.167	0.095	*0212400*	DEL
5	1103780	67	0.415	0.293	0.600	0.512	0.396	0.330	0.042	0.136	*0526600*	DEL
11	294600	26	0.410	0.264	0.236	0.296	0.170	0.499	0.375	0.053	*1107100*	DEL
2	823170	477	0.407	0.001	0.003	0.000	0.509	0.774	0.125	0.502		DEL
12	1153072	23	0.407	0.255	0.200	0.265	0.321	0.517	0.000	0.122		DEL
5	217388	28	0.404	0.356	0.733	0.644	0.302	0.200	0.208	0.179	*0505000*	DEL
2	846149	27	0.403	0.482	0.542	0.512	0.396	0.218	0.417	0.046		DEL
5	207278	31	0.401	0.256	0.317	0.302	0.377	0.461	0.125	0.051	*0504900*	DEL
13	1030635	145	0.401	0.241	0.414	0.381	0.623	0.409	0.167	0.089	*1324600*	DEL
13	1683099	19	0.399	0.316	0.306	0.348	0.132	0.422	0.125	0.061	*1342900*	DEL
14	303131	43	0.399	0.224	0.519	0.428	0.434	0.379	0.083	0.096	*1408200*	DEL
9	1437364	61	0.398	0.159	0.267	0.340	0.623	0.492	0.208	0.116	*0936300*	DEL
7	1035250	27	0.392	0.243	0.389	0.418	0.358	0.394	0.167	0.038	*0724700*	DEL
7	1174576	41	0.392	0.199	0.247	0.210	0.208	0.522	0.292	0.063		DEL
4	1000639	29	0.390	0.398	0.467	0.467	0.189	0.281	0.167	0.069		DEL
5	498679	19	0.387	0.329	0.303	0.278	0.245	0.409	0.292	0.012	*0511500*	DEL

10	111397	83	0.384	0.091	0.386	0.543	0.321	0.416	0.083	0.134		DEL
3	343910	16	0.378	0.519	0.472	0.475	0.075	0.196	0.208	0.133	*0307900*	DEL
3	537167	57	0.376	0.000	0.000	0.000	0.698	0.709	0.000	0.613	*0312900*	DEL
7	453367	32	0.368	0.253	0.353	0.247	0.151	0.411	0.333	0.035	*0710000*	DEL
3	889903	49	0.365	0.095	0.194	0.249	0.377	0.520	0.000	0.163		DEL
13	2340012	25	0.365	0.264	0.453	0.449	0.245	0.309	0.000	0.113	*1359000*	DEL
7	384674	27	0.364	0.222	0.256	0.074	0.264	0.502	0.000	0.148		DEL
1	426936	19	0.363	0.424	0.550	0.531	0.415	0.164	0.292	0.075	*0111000*	DEL
10	1260592	25	0.363	0.007	0.033	0.023	0.396	0.675	0.083	0.386	*1031300*	DEL
8	1255382	23	0.360	0.286	0.358	0.383	0.321	0.328	0.250	0.009		DEL
6	326953	37	0.359	0.221	0.419	0.393	0.396	0.339	0.083	0.068	*0607700*	DEL
7	120934	33	0.359	0.377	0.136	0.447	0.094	0.322	0.208	0.084		DEL
7	454369	31	0.359	0.260	0.317	0.292	0.264	0.378	0.417	0.016	*0710000*	DEL
8	1248470	61	0.355	0.184	0.200	0.270	0.434	0.444	0.125	0.075	*0829000*	DEL
6	487816	33	0.354	0.118	0.236	0.290	0.283	0.468	0.000	0.120		DEL
6	940032	31	0.354	0.184	0.428	0.393	0.321	0.348	0.125	0.057	*0623100*	DEL
7	144688	49	0.354	0.234	0.253	0.278	0.415	0.402	0.083	0.062	*0703600*	DEL
11	352801	25	0.353	0.126	0.156	0.093	0.528	0.537	0.125	0.193	*1108000*	DEL

SEA = Southeast Asia; DUP = duplication; DEL = deletion

Supplementary table 5

High quality common variants greater than 300 bp in length from the window-based analysis

Chr.	Start	End	*Gene*	Name	Type	Freq.	West Africa	Central Africa	East Africa	South Asia	SEA	South America	Regional F_{ST}
2	735000	736000	*PF3D7_0217900*		DEL	54	0.022	0.017	0.049	0.000	0.005	0.000	0.019
2	797500	799500	*PF3D7_0220000*	*LSA3*	DEL	156	0.001	0.000	0.002	0.038	0.104	0.000	0.062
2	822500	824500	*PF3D7_0220500*		DEL	1163	0.001	0.003	0.000	0.509	0.774	0.125	0.502
3	512000	513000	*PF3D7_0312000*		DEL	42	0.008	0.003	0.004	0.000	0.023	0.000	0.010
4	743000	744000	*PF3D7_0417000/100*		DEL	166	0.060	0.047	0.072	0.000	0.048	0.000	0.022
5	125000	126000	*PF3D7_0503000*		DEL	80	0.037	0.017	0.064	0.019	0.010	0.000	0.018
5	184500	186500	*PF3D7_0504700*	*CEP120*	DEL	92	0.000	0.003	0.000	0.019	0.060	0.125	0.062
5	339000	341000	*PF3D7_0508200*	*LAG1*	DEL	46	0.033	0.000	0.019	0.000	0.009	0.000	0.015
5	728500	730500			DEL	116	0.070	0.000	0.014	0.000	0.036	0.167	0.075
8	189000	190500	*PF3D7_0802700*		DEL	44	0.026	0.017	0.029	0.019	0.002	0.042	0.007
8	425000	427000	*PF3D7_0808450*		DEL	155	0.089	0.017	0.062	0.000	0.035	0.083	0.024
8	1025500	1027500			DEL	37	0.003	0.000	0.002	0.000	0.023	0.000	0.015
9	1318000	1320000	*PF3D7_0933200*		DEL	49	0.011	0.000	0.014	0.019	0.023	0.000	0.007
9	1384000	1385000			DEL	85	0.000	0.000	0.000	0.000	0.058	0.000	0.049
9	1384500	1386000			DEL	355	0.007	0.011	0.002	0.000	0.236	0.000	0.183
9	1385500	1386500			DEL	322	0.003	0.008	0.000	0.000	0.217	0.000	0.176
9	1388500	1390000			DEL	43	0.005	0.011	0.039	0.000	0.010	0.083	0.035
9	1397000	1399500			DEL	1233	0.115	0.294	0.039	0.094	0.685	0.708	0.348
10	110000	112000			DEL	287	0.004	0.058	0.187	0.151	0.112	0.000	0.064
10	1043000	1045000	*PF3D7_1024900*		DEL	199	0.000	0.000	0.000	0.000	0.136	0.000	0.116
11	1055000	1056500	*PF3D7_1127000*		DEL	160	0.070	0.214	0.064	0.000	0.000	0.000	0.106
11	1842000	1843500	*PF3D7_1146700*		DEL	124	0.054	0.053	0.039	0.000	0.031	0.042	0.009
12	575000	576000	*PF3D7_1213000*		DEL	1015	0.001	0.000	0.000	0.472	0.677	0.000	0.496
12	716500	718500	*PF3D7_1218300*	*AP2-MU*	DEL	511	0.000	0.000	0.006	0.075	0.345	0.000	0.239

12	924500	926500	*PF3D7_1223000/2900*		DEL	47	0.003	0.003	0.091	0.000	0.000	0.000	0.071
12	974000	975000	*PF3D7_1224000*	*GCH1*	DUP	491	0.068	0.172	0.780	0.000	0.000	0.000	0.554
12	1030000	1031500	*PF3D7_1225300*		DEL	627	0.180	0.183	0.119	0.264	0.235	0.542	0.099
12	1031000	1032000	*PF3D7_1225400/300*		DEL	627	0.180	0.183	0.119	0.264	0.235	0.542	0.099
13	403000	404000	*PF3D7_1308700/800*		DEL	80	0.003	0.003	0.002	0.000	0.052	0.000	0.036
13	754000	756000	*PF3D7_1318300*		DEL	37	0.026	0.000	0.010	0.000	0.008	0.042	0.016
13	2052000	2054000	*PF3D7_1351500/400*		DEL	30	0.009	0.003	0.010	0.019	0.011	0.000	0.004
13	2097000	2099000	*PF3D7_1352600*		DEL	157	0.043	0.047	0.076	0.019	0.048	0.000	0.015
13	2561000	2563000	*PF3D7_1363900*		DEL	1341	0.566	0.411	0.459	0.302	0.359	0.458	0.028
14	757000	758500	*PF3D7_1417900*		DEL	566	0.205	0.225	0.245	0.283	0.134	0.167	0.015
14	2760500	2762500	*PF3D7_1467600*		DEL	37	0.008	0.006	0.014	0.000	0.015	0.000	0.005

SEA = Southeast Asia; DUP = duplication; DEL = deletion

Supplementary table 6

An analysis of *crt* duplications and resistance haplotypes in the West African isolates

crt haplotype	Duplication	No duplication
CVIET	0 [1]	201 [214]
CVMNK	0 [1]	322 [343]
CVIET + CVMNK	35 [33]*	133 [99]
	35	656

CVMNK = chloroquine susceptible; CVIET = chloroquine resistant; the table shows the number of West African isolates with >0 read support or a sensitivity analysis where >1 read support was required [square brackets] to call haplotypes; * Burkina Faso (n=15), Ghana (n=18), Guinea (n=1), Mali (n=1); note, there were two Cambodian samples with evidence of a duplication

Supplementary table 7

Most distinct variants (all sizes) by region-based F_{ST} values

Chr.	Start	Length	Freq.	West Africa	Central Africa	East Africa	South Asia	SEA	South America	Regional F_{ST}	Gene (*PF3D7_*)	Model
3	537167	57	0.376	0.000	0.000	0.000	0.698	0.709	0.000	0.613	*0312900*	DEL
12	973804	436	0.172	0.068	0.172	0.780	0.000	0.000	0.000	0.554		DUP
6	917407	38	0.005	0.000	0.000	0.000	0.000	0.000	0.583	0.538		DEL
2	823170	477	0.407	0.001	0.003	0.000	0.509	0.774	0.125	0.502		DEL
14	2947728	40	0.005	0.000	0.000	0.000	0.000	0.000	0.542	0.497	*1472200*	DEL
12	230336	25	0.017	0.011	0.008	0.043	0.019	0.001	0.583	0.454		DEL
14	2634017	33	0.027	0.014	0.022	0.043	0.000	0.016	0.583	0.442		DEL
2	616052	35	0.353	0.016	0.003	0.025	0.245	0.663	0.000	0.436		DEL
6	817854	20	0.008	0.003	0.000	0.004	0.019	0.003	0.500	0.422	*0619400*	DEL
4	1091332	47	0.033	0.000	0.000	0.000	0.509	0.045	0.000	0.418		DEL
14	2062213	37	0.310	0.007	0.011	0.012	0.453	0.580	0.000	0.404	*1450400*	DEL
5	1192722	39	0.505	0.125	0.200	0.095	0.604	0.821	0.042	0.396		DEL
10	1260592	25	0.363	0.007	0.033	0.023	0.396	0.675	0.083	0.386	*1031300*	DEL
11	252507	35	0.010	0.018	0.006	0.004	0.000	0.001	0.458	0.381	*1105800*	DEL
14	1454153	21	0.028	0.034	0.022	0.068	0.000	0.001	0.542	0.381		DEL
13	1205937	88	0.259	0.001	0.011	0.000	0.075	0.500	0.000	0.374	*1328500*	DEL
7	412301	25	0.004	0.000	0.000	0.000	0.000	0.000	0.417	0.373		DEL
12	510059	23	0.004	0.001	0.003	0.000	0.000	0.000	0.417	0.369		DEL
5	424537	34	0.280	0.004	0.000	0.002	0.377	0.530	0.000	0.367	*0510100*	DEL
13	1489977	23	0.005	0.003	0.003	0.000	0.000	0.000	0.417	0.366	*1336900*	DEL
10	123144	36	0.025	0.038	0.047	0.019	0.038	0.001	0.542	0.364	*1002400*	DEL
13	1000877	28	0.036	0.019	0.006	0.002	0.075	0.047	0.542	0.364	*1324200*	DEL
10	484230	23	0.006	0.005	0.000	0.004	0.000	0.000	0.417	0.363		DEL
6	1117926	31	0.071	0.000	0.006	0.412	0.000	0.000	0.000	0.362		DEL
11	109717	31	0.032	0.042	0.017	0.033	0.038	0.016	0.542	0.361		DEL
11	1094625	27	0.277	0.004	0.014	0.004	0.415	0.520	0.000	0.361		DEL
14	572110	21	0.014	0.003	0.006	0.010	0.019	0.013	0.458	0.359		DEL
12	1391604	31	0.007	0.011	0.000	0.004	0.000	0.001	0.417	0.355	*1233600*	DEL
5	626767	39	0.269	0.003	0.000	0.002	0.189	0.517	0.000	0.349		DEL
12	574590	420	0.270	0.000	0.000	0.000	0.302	0.517	0.000	0.349		DEL
13	2015123	41	0.334	0.003	0.028	0.000	0.226	0.634	0.167	0.339		DEL
13	2409230	35	0.253	0.000	0.006	0.002	0.132	0.487	0.000	0.337		DEL
10	1133623	68	0.278	0.015	0.008	0.006	0.283	0.522	0.000	0.33		DEL
2	218932	35	0.005	0.005	0.003	0.000	0.000	0.000	0.375	0.324		DEL
10	989500	37	0.005	0.000	0.000	0.008	0.000	0.000	0.375	0.324	*1023600*	DEL
4	420121	23	0.006	0.007	0.000	0.006	0.000	0.000	0.375	0.319	*0408700*	DEL
9	1159735	49	0.007	0.004	0.000	0.004	0.000	0.005	0.375	0.319		DEL
14	1443359	23	0.251	0.001	0.003	0.000	0.264	0.479	0.000	0.315		DEL

11	1151552	45	0.011	0.007	0.000	0.002	0.113	0.005	0.458	0.314		DEL
13	1882747	27	0.014	0.011	0.017	0.031	0.000	0.001	0.417	0.313		DEL
6	462990	31	0.010	0.024	0.000	0.002	0.000	0.000	0.375	0.306		DEL
8	1192964	82	0.282	0.022	0.019	0.033	0.358	0.511	0.000	0.306		DEL
11	737028	37	0.490	0.171	0.150	0.158	0.509	0.763	0.042	0.305	*1119500*	DEL
14	1358459	29	0.018	0.000	0.000	0.000	0.000	0.028	0.375	0.304		DEL
11	315057	29	0.208	0.000	0.003	0.002	0.057	0.402	0.000	0.301		DEL
13	2312787	25	0.559	0.247	0.250	0.329	0.679	0.772	0.000	0.301	*1358200*	DEL
9	1266743	30	0.201	0.019	0.011	0.008	0.000	0.379	0.000	0.297		DEL
7	1190837	31	0.220	0.003	0.003	0.014	0.075	0.421	0.000	0.294		DEL
6	548419	25	0.445	0.103	0.158	0.140	0.472	0.714	0.042	0.293	*0613500*	DEL
14	735240	25	0.003	0.001	0.000	0.000	0.000	0.000	0.333	0.293	*1417600*	DEL
7	868789	34	0.229	0.003	0.003	0.000	0.151	0.440	0.000	0.292	*0719900*	DEL
6	421332	25	0.004	0.000	0.000	0.000	0.000	0.002	0.333	0.291	*0609800*	DEL
7	838837	29	0.003	0.000	0.000	0.002	0.000	0.000	0.333	0.291		DEL
6	350115	25	0.031	0.042	0.047	0.049	0.000	0.003	0.458	0.29	*0608400*	DEL
12	1025776	37	0.204	0.194	0.364	0.609	0.075	0.006	0.000	0.29	*1225200*	DEL
3	537150	29	0.271	0.328	0.628	0.562	0.019	0.020	0.167	0.286	*0312900*	DEL
7	1151359	31	0.276	0.030	0.056	0.041	0.075	0.495	0.000	0.285		DEL
9	243657	42	0.487	0.180	0.217	0.154	0.472	0.738	0.000	0.284		DEL
6	974085	25	0.005	0.003	0.003	0.004	0.000	0.000	0.333	0.282		DEL
8	968599	31	0.264	0.018	0.053	0.021	0.377	0.474	0.000	0.28	*0821500*	DEL
13	996382	32	0.224	0.001	0.000	0.000	0.226	0.428	0.000	0.279		DEL
4	249735	25	0.438	0.709	0.753	0.644	0.189	0.081	0.625	0.278	*0404600*	DEL
9	1397960	101<br>5	0.301	0.115	0.292	0.039	0.075	0.430	0.708	0.278		DEL
3	612411	23	0.025	0.030	0.017	0.056	0.000	0.004	0.417	0.277		DEL
9	749680	37	0.198	0.016	0.011	0.021	0.434	0.354	0.000	0.275	*0918200*	DEL
9	537684	49	0.257	0.015	0.044	0.029	0.377	0.461	0.000	0.274		DEL
7	938664	23	0.022	0.038	0.036	0.014	0.019	0.003	0.417	0.272	*0721800*	DEL
13	553010	30	0.161	0.003	0.000	0.000	0.000	0.313	0.000	0.272		DEL
1	349483	45	0.211	0.000	0.003	0.000	0.302	0.400	0.000	0.271	*0108500*	DEL
9	1280866	19	0.302	0.072	0.031	0.041	0.434	0.517	0.042	0.271	*0932100*	DEL
14	887749	30	0.325	0.023	0.036	0.051	0.226	0.588	0.125	0.27	*1421600*	DEL
12	1583456	57	0.435	0.122	0.122	0.193	0.396	0.679	0.000	0.269		DEL
6	329851	31	0.017	0.012	0.003	0.019	0.019	0.013	0.375	0.267	*0607800*	DEL
14	3191550	29	0.018	0.037	0.017	0.014	0.000	0.002	0.375	0.266		DEL
13	663645	27	0.008	0.005	0.014	0.008	0.000	0.001	0.333	0.264		DEL
12	229065	27	0.214	0.003	0.000	0.006	0.170	0.408	0.000	0.261		DEL
13	436314	33	0.235	0.020	0.011	0.027	0.113	0.434	0.000	0.26		DEL
5	992729	21	0.052	0.041	0.136	0.119	0.000	0.000	0.500	0.259		DEL
8	557474	23	0.180	0.001	0.017	0.463	0.057	0.191	0.000	0.259		DEL
12	217330	41	0.006	0.008	0.006	0.000	0.019	0.000	0.333	0.259	*1204900*	DEL

14	2838402	23	0.163	0.004	0.006	0.004	0.000	0.313	0.000	0.259		DEL
14	2185959	37	0.195	0.014	0.006	0.002	0.057	0.370	0.000	0.257	*1453200*	DEL
2	148195	38	0.002	0.000	0.000	0.000	0.000	0.000	0.292	0.256		DEL
10	249371	27	0.002	0.000	0.000	0.000	0.000	0.000	0.292	0.256	*1005700*	DEL
14	1168107	29	0.002	0.000	0.000	0.000	0.000	0.000	0.292	0.256		DEL
10	834621	24	0.003	0.001	0.000	0.000	0.000	0.000	0.292	0.255		DEL
14	1948787	22	0.003	0.001	0.000	0.000	0.000	0.000	0.292	0.255	*1447800*	DEL
3	453754	34	0.223	0.001	0.006	0.002	0.415	0.415	0.125	0.254	*0310500*	DEL
8	965383	46	0.056	0.084	0.053	0.132	0.019	0.001	0.500	0.254		DEL
7	695261	22	0.003	0.001	0.000	0.002	0.000	0.000	0.292	0.252		DEL
14	1707194	19	0.011	0.018	0.014	0.008	0.000	0.000	0.333	0.252	*1442100*	DEL
10	486850	23	0.011	0.012	0.019	0.008	0.000	0.002	0.333	0.251		DEL
14	955192	24	0.222	0.003	0.008	0.000	0.302	0.420	0.042	0.251		DEL
8	680554	55	0.004	0.001	0.000	0.004	0.000	0.000	0.292	0.25		DEL
9	907996	20	0.075	0.005	0.036	0.391	0.075	0.003	0.000	0.25	*0922300*	DEL
14	529549	37	0.332	0.033	0.069	0.064	0.377	0.578	0.125	0.246		DEL
10	1468745	37	0.201	0.001	0.000	0.008	0.264	0.379	0.000	0.245	*1037000*	DEL
6	1015998	39	0.278	0.037	0.044	0.051	0.208	0.490	0.000	0.244	*0624900*	DEL
10	635151	21	0.005	0.001	0.003	0.008	0.000	0.000	0.292	0.242	*1015800*	DEL
6	1214164	33	0.005	0.001	0.006	0.006	0.000	0.000	0.292	0.241	*0629500*	DEL

SEA = Southeast Asia; DUP = duplication; DEL = deletion
